# Improvement of cellulose catabolism in *Clostridium cellulolyticum* by sporulation abolishment and carbon alleviation

**DOI:** 10.1186/1754-6834-7-25

**Published:** 2014-02-20

**Authors:** Yongchao Li, Tao Xu, Timothy J Tschaplinski, Nancy L Engle, Yunfeng Yang, David E Graham, Zhili He, Jizhong Zhou

**Affiliations:** 1 Institute for Environmental Genomics and Department of Microbiology and Plant Biology, University of Oklahoma, 101 David L. Boren Boulevard, Norman, OK 73019, USA; 2 BioEnergy Science Center, Oak Ridge National Laboratory, Oak Ridge, TN 37831, USA; 3 State Key Joint Laboratory of Environment Simulation and Pollution Control, School of Environment, Tsinghua University, Beijing 100084, China; 4 Biosciences Division, Oak Ridge National Laboratory, Oak Ridge, TN 37831, USA; 5 Earth Sciences Division, Lawrence Berkeley National Laboratory, Berkeley, CA 94720, USA

**Keywords:** *Clostridium cellulolyticum*, Sporulation, *spo0A*, Cellulose catabolism, Isobutanol

## Abstract

**Background:**

*Clostridium cellulolyticum* can degrade lignocellulosic biomass, and ferment the soluble sugars to produce valuable chemicals such as lactate, acetate, ethanol and hydrogen. However, the cellulose utilization efficiency of *C. cellulolyticum* still remains very low, impeding its application in consolidated bioprocessing for biofuels production. In this study, two metabolic engineering strategies were exploited to improve cellulose utilization efficiency, including sporulation abolishment and carbon overload alleviation.

**Results:**

The *spo0A* gene at locus *Ccel_1894*, which encodes a master sporulation regulator was inactivated. The *spo0A* mutant abolished the sporulation ability. In a high concentration of cellulose (50 g/l), the performance of the *spo0A* mutant increased dramatically in terms of maximum growth, final concentrations of three major metabolic products, and cellulose catabolism. The microarray and gas chromatography–mass spectrometry (GC-MS) analyses showed that the valine, leucine and isoleucine biosynthesis pathways were up-regulated in the *spo0A* mutant. Based on this information, a partial isobutanol producing pathway modified from valine biosynthesis was introduced into *C. cellulolyticum* strains to further increase cellulose consumption by alleviating excessive carbon load. The introduction of this synthetic pathway to the wild-type strain improved cellulose consumption from 17.6 g/l to 28.7 g/l with a production of 0.42 g/l isobutanol in the 50 g/l cellulose medium. However, the *spo0A* mutant strain did not appreciably benefit from introduction of this synthetic pathway and the cellulose utilization efficiency did not further increase. A technical highlight in this study was that an *in vivo* promoter strength evaluation protocol was developed using anaerobic fluorescent protein and flow cytometry for *C. cellulolyticum*.

**Conclusions:**

In this study, we inactivated the *spo0A* gene and introduced a heterologous synthetic pathway to manipulate the stress response to heavy carbon load and accumulation of metabolic products. These findings provide new perspectives to enhance the ability of cellulolytic bacteria to produce biofuels and biocommodities with high efficiency and at low cost directly from lignocellulosic biomass*.*

## Background

As the search for affordable and clean energy fuels continues, cellulosic biofuels have become a promising solution because cellulosic biomass is the most abundant renewable feedstock on earth [1]. The key technological barrier to utilize this important renewable resource is the general lack of low-cost technology for overcoming the recalcitrance of cellulosic biomass on a large-scale [2]. Consolidated bioprocessing (CBP), which integrates saccharolytic enzymes production, cellulose fiber degradation, and fermentation of resulting sugars into a single step, is considered a promising technology for significantly reducing the processing cost [3].

*Clostridium cellulolyticum* is a mesophilic gram-positive bacterium capable of degrading cellulose via an extracellular enzymatic complex called the cellulosome and fermenting the sugars from cellulose degradation to lactate, acetate, ethanol, hydrogen and CO_2_[4]. Recently, *C. cellulolyticum* was engineered to produce isobutanol, a possible alternative to gasoline to fuel combustion engines, directly from cellulose [5]. Therefore, *C. cellulolyticum* has the potential to be a model system with industrial relevance for the production of biofuels and commodity chemicals directly from plant biomass via CBP. However, its cellulolytic ability and metabolic productivity still need to be improved dramatically in order to meet the requirements of industrial applications. Enzymological properties of the *C. cellulolyticum* cellulosome have been studied extensively and there is no evidence showing that its cellulolytic system is a limiting factor for cellulose utilization [4]. Therefore, other metabolic engineering strategies need to be exploited to improve the cellulose utilization efficiency.

Hydrolysis of lignocellulose at high concentrations is essential in economical fermentation to ethanol and other valuable products. Also, there are advantages if the metabolic products can be accumulated at high concentrations to reduce the cost of product recovery and lower energy input [6]. However, in nature *C. cellulolyticum* can rarely find a niche where carbon sources and all other nutrients are plentiful, so it has become well adapted for famine environments after millions of years of evolution [7]. These natural ecosystems are quite different from the fermentation conditions in the laboratory or industrial sites where most nutrient factors have been optimized. Indeed, it has been reported that *C. cellulolyticum* could not deal with a surfeit of substrates, leading to nicotinamide adenine dinucleotide (NADH) and pyruvate accumulation to toxic levels [8]. This problem was alleviated by heterologous expression of the *Zymomonas mobilis* pyruvate decarboxylase and alcohol dehydrogenase genes to reduce pyruvate accumulation. As a consequence, cellulose consumption was increased 150% compared to the wild-type (WT) [9]. Meanwhile, it has been reported that high concentration of cellulose and low pH could trigger sporulation and the entry into the stationary phase of *C. cellulolyticum*, which may be partially responsible for arresting metabolite production [10]. It has been noticed that attachment to cellulose fibers could trigger sporulation in *Clostridium thermocellum*[11], indicating a possible connection between sporulation and cellulose degradation. It also has been recognized that the sporulation program could be a hindrance for applying sporulating microbes in fermentations for commodity chemicals production [12]. Since sporulation is not a desirable trait from an industrial point of view in solventogenic *Clostridium acetobutylicum*, it is of particular importance to investigate how the transcriptional regulation of sporulation impacts solventogenesis. Several key sporulation-related transcriptional regulator genes were inactivated, and the interconnections between differentiation, sporulation and solventogenesis were studied extensively in recent years [13-15].

Motivated by the work mentioned above, we investigated approaches based on manipulating the stress response caused by heavy carbon loading, accumulation of metabolic products, and accompanied physiological changes. Sporulation is a widely used strategy by gram-positive bacteria to increase survival ability in hostile environments by entering a dormant or non-growth state, and forming a robust spore that germinates when conditions are favorable [16]*.* In *C. cellulolyticum*, fermentation causes acids accumulation resulting in a drop in pH and accumulation of various metabolic products. Such accumulations can create a less favorable growth condition for *C. cellulolyticum*, which could trigger the sporulation process and slow down cellulose hydrolysis and metabolism. Therefore, by curbing sporulation in *C. cellulolyticum*, the fermentation process might be extended to improve cellulose hydrolysis and metabolite production. Furthermore, carbon overload could be alleviated by introducing exogenous pyruvate consumption pathways to further improve cellulose hydrolysis. To test these hypotheses, we disrupted the *spo0A* gene at locus *Ccel_1894* encoding the master-switch transcription factor of sporulation [17], and introduced a synthetic isobutanol pathway [18] into WT and *spo0A* mutant strains to consume excessive pyruvate. Results with strains designed to test these hypotheses are reported in this study.

## Results

### Group II intron-mediated *spo0A* inactivation

**Figure 1:**
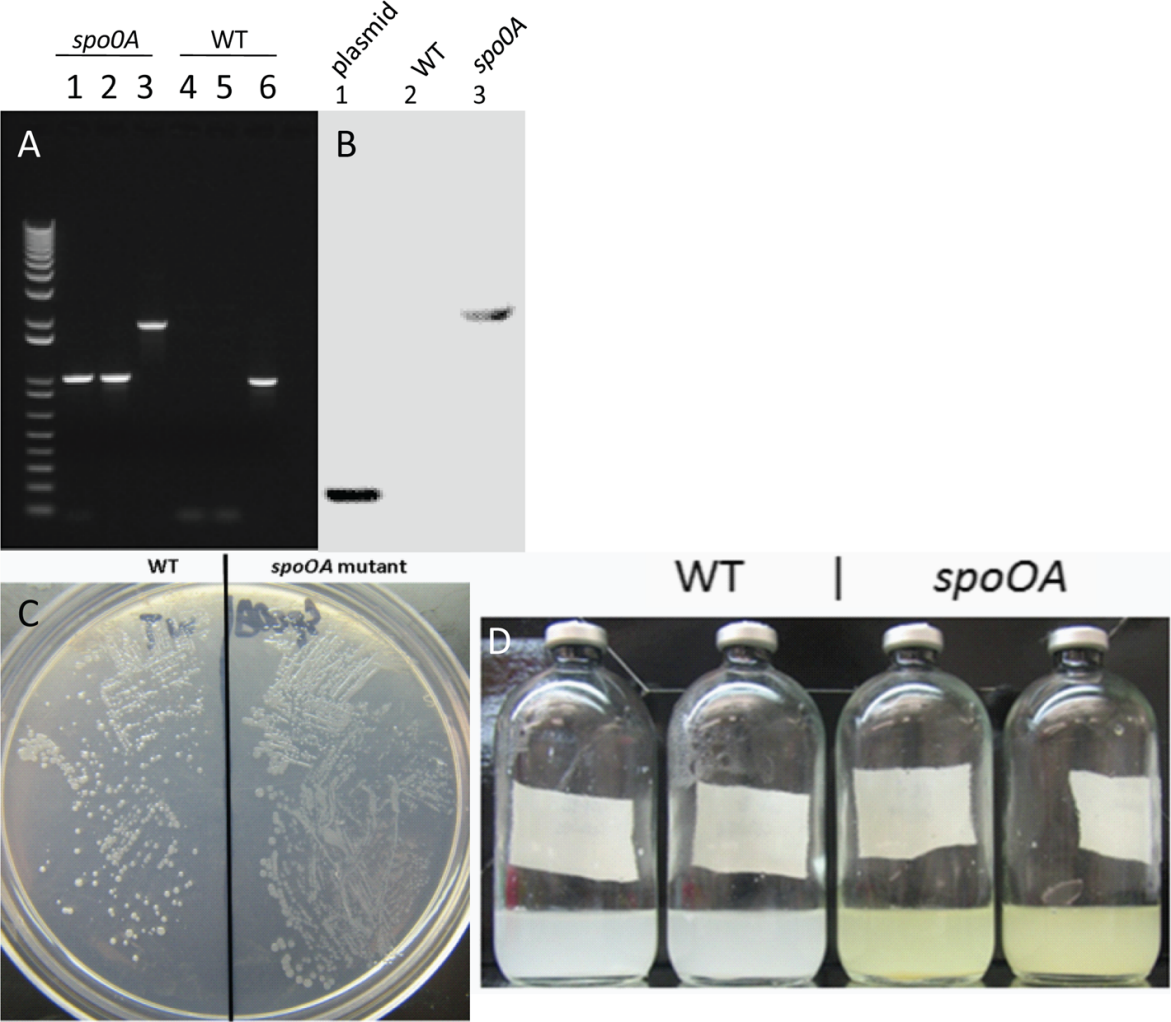
**Generation and characterization of the *spo0A *mutant. (A)** Confirmation of pure culture of the *spo0A* mutant by PCR, using different combinations of primers. Amplification of intron-*spo0A* junction regions using one primer in the genome and the other in the intron (Spo0AF/intronF1 and intronR1/Spo0AR) resulted in bands from the *Ccel_1894* mutant (lanes 1 and 2), but not in wild-type (WT) cells (lanes 4 and 5). In PCR reactions using Spo0AF/Spo0AR primers, the mutant showed a single band (lane 3), which was 915 bp larger than the single band (lane 6) in WT cells, confirming the expected intron insertion. **(B)** Southern blot with biotin-labeled intron-specific probes showing a single intron insertion band for *spo0A* mutant (lane 3), and no band for WT (lane 2). The intron donor plasmid was used as a positive control (lane 1). **(C)** Agar plate showing that the colonial morphology of *spo0A* mutant (right) was flatter and more translucent compared to WT (left). **(D)** Comparison of cellulose fermentation broth showing that *spo0A* mutant culture produced a yellow-green pigment (right), which was not observed for WT (left).

*C. cellulolyticum* cells transformed with pLyc1217Er-based vectors yielded erythromycin-resistant colonies on agar plates. Colony PCR was performed using forward and reverse primers flanking the intron insertion sites of *Ccel_1894* to screen transformants for the desired insertion. To further confirm the correct insertion sites and intron orientation of the isolated mutant strains, colony PCR was performed using combinations of forward and reverse primers flanking the target genes and intron-specific primers (Figure 1A). A Southern blot was performed on genomic DNA from the *Ccel_1894* mutant using an intron-specific probe (Figure 1B). The mutant contained a single intron insertion in the chromosome with expected size of 4.2 kbp. The knockout plasmid pLyc1217Er showed a band of 1.5 kbp. No band was detected in chromosomal DNA from WT cells. Finally, the PCR product from the mutant amplified by primers Spo0AF/Spo0AR was sequenced, verifying the correct intron insertion in the mutant strain.

### Characterization of *spo0A* mutant

**Figure 2:**
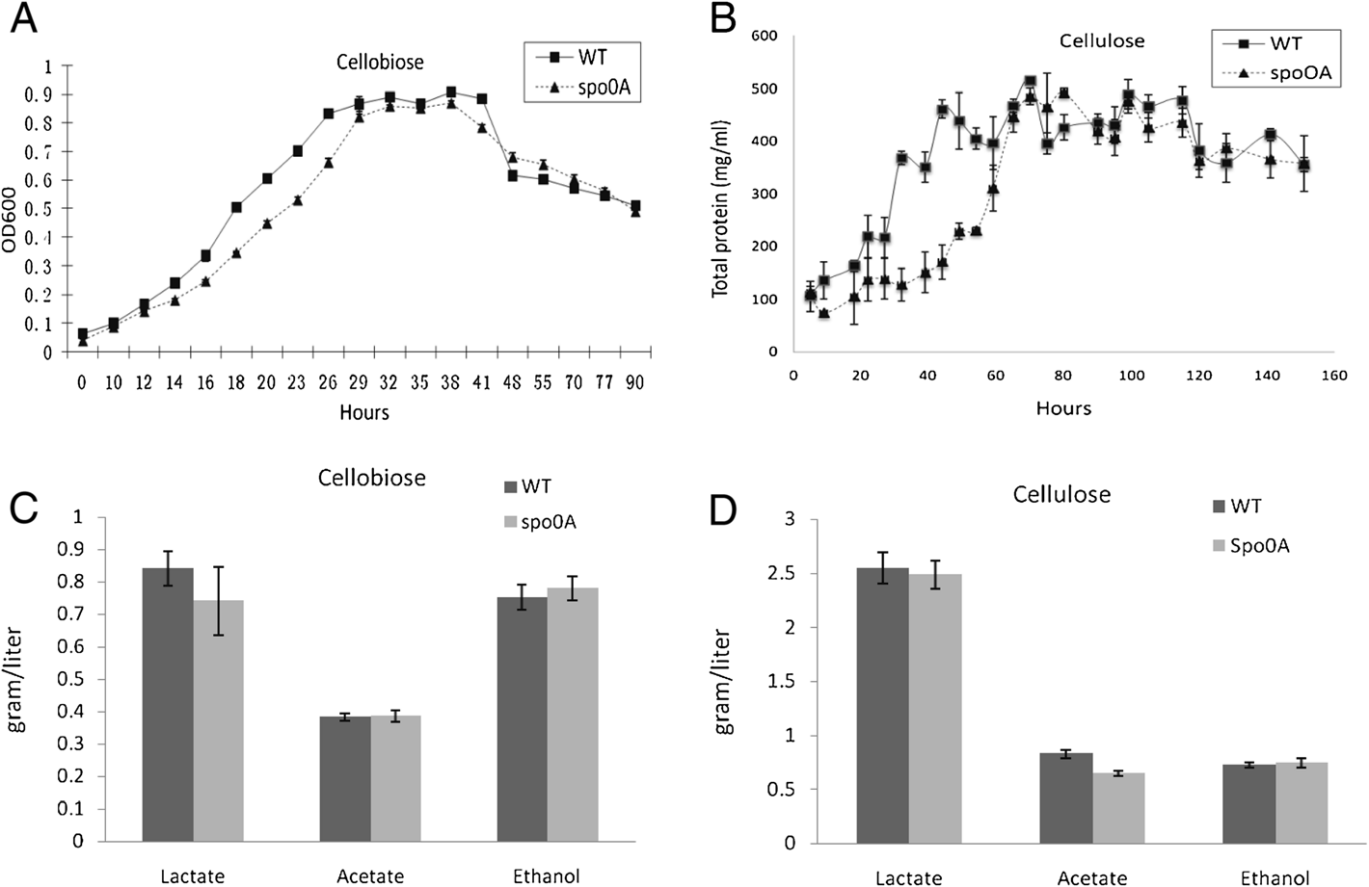
**Growth curves and maximum production of lactate, acetate and ethanol by *C. cellulolyticum *strains. (A)** Growth measured by optical density at 600 nm (OD_600_) in 5 g/l cellobiose. Wild-type (WT) and *spo0A* mutant reached a similar maximum growth, and *spo0A* mutant had a slightly lagged log phase. **(B)** Growth measured by total cellular protein in 10 g/l cellulose. WT and *spo0A* mutant reached a similar maximum growth, and *spo0A* mutant had a more obvious lagged log phase. **(C and D)** Final metabolic product concentrations measured for WT and *spo0A* mutant in 5 g/l cellobiose and 10 g/l cellulose, respectively. In both of the carbon sources, the WT and *spo0A* mutant produced similar amounts of lactate, acetate and ethanol. The error bars represent standard deviations of measurements from three replicate cultures.

**Figure 3:**
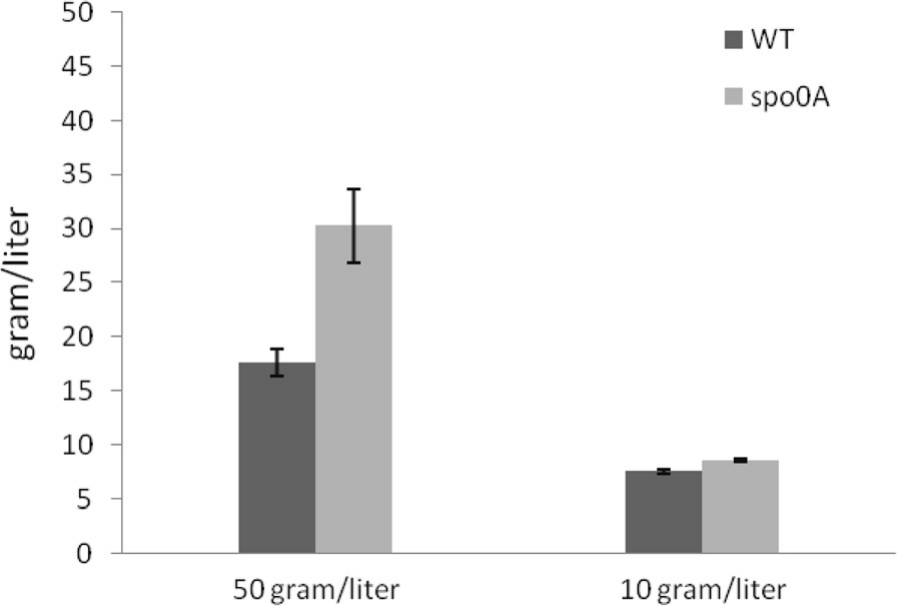
**Total cellulose utilization by wild-type (WT) and *spo0A *mutant grown in 50 g/l and 10 g/l cellulose, respectively.** The fermentation time was 256 hours with 50 g/l and 156 hours with 10 g/l cellulose, respectively. The error bars represent standard deviations of measurements from three replicate cultures.

**Figure 4:**
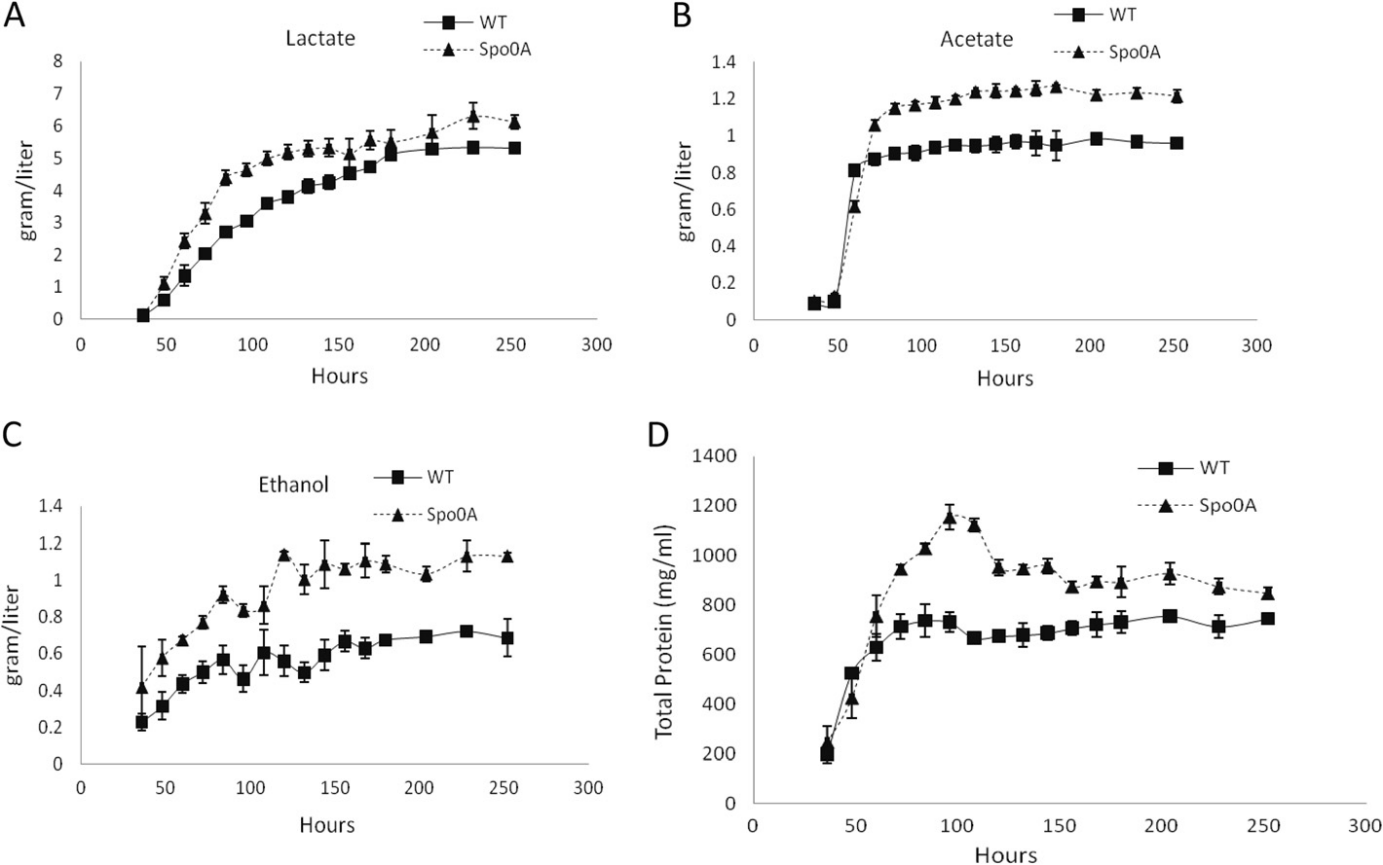
**Production profiles of lactate, acetate and ethanol and growth curves of *C. cellulolyticum *strains in 50 g/l cellulose.** All of the three major metabolic products, lactate **(A)**, acetate **(B)**, and ethanol **(C)** were increased in *spo0A* mutant, with the highest increase in ethanol production. The major metabolic product of both strains was lactate. The growth of *spo0A* mutant **(D)** was increased 53% in the total cellular biomass, but the total cellular biomass of *spo0A* mutant dropped more rapidly than WT after reaching the peak growth. The error bars represent standard deviations of measurements from three replicate cultures.

It was previously reported that in an unregulated-pH cellulose medium, *C. cellulolyticum* could reach a sporulation rate of 45% [10]. To allow enough sporulation, the strains were grown in cellulose medium for 18 days before heat shock and plating. Sporulation, as tested by sporulation heat survival assay, was observed in WT strain (35.7 ± 7.3%) and in the complementary Spo0A/over strain (26.0% ± 2.8%). However, no detectable colonies formed after heat shock of the *spo0A* mutant and the *spo0A* mutant holding the empty vector. Isolated colonies formed by the *spo0A* mutant were flatter and more translucent, which were similar in morphology to previously reported *spo0A* mutants from several *Bacillus* and *Clostridium* species [19-21] (Figure 1C). In the defined cellulose medium, the mutant culture was yellowish, which was not observed in the WT culture (Figure 1D), although we could not identify the source of the yellow pigment. With low concentrations of carbon sources (5 g/l cellobiose or 10 g/l cellulose), there was no obvious growth defect detected for the *spo0A* mutant (Figure 2A and 2B), as reflected by OD_600_ measurement in cellobiose and total pellet protein measurement in cellulose, respectively. The mutant reached a similar maximum growth to the WT culture, but with a slight delay in the exponential phase. The concentrations of the three major metabolic products, lactate, acetate, and ethanol, were not significantly different between *spo0A* mutant and WT after five days fermentation with 5 g/l cellobiose or after 10 days fermentation with 10 g/l cellulose (Figure 2C and 2D). The data were consistent with the *spo0A* mutant of *C. beijerinckii*[21], but different from the *C. acetobutylicum spo0A* mutant, which produced 75% less ethanol than WT cells [22]. WT utilized 7.56 g/l cellulose, and *spo0A* mutant utilized 8.60 g/l (Figure 3). To examine whether sporulation abolishment would affect high-concentration cellulose catabolism, we increased the cellulose load to 50 g/l. The performance of the *spo0A* mutant increased dramatically in the high concentration of cellulose. The concentrations of three major metabolic products were all increased in the *spo0A* mutant, with ethanol increased the most (Figure 4A,B and C), and the maximum growth of the *spo0A* mutant increased by 53% (Figure 4D). Accordingly, the cellulose utilization was also increased dramatically in the *spo0A* mutant, which was 72% higher than the WT with the final cellulose catabolism of 30.3 g out of the 50 g (Figure 3).

### Transcriptomic and metabolomic comparison of WT and *spo0A* mutant

**Figure 5:**
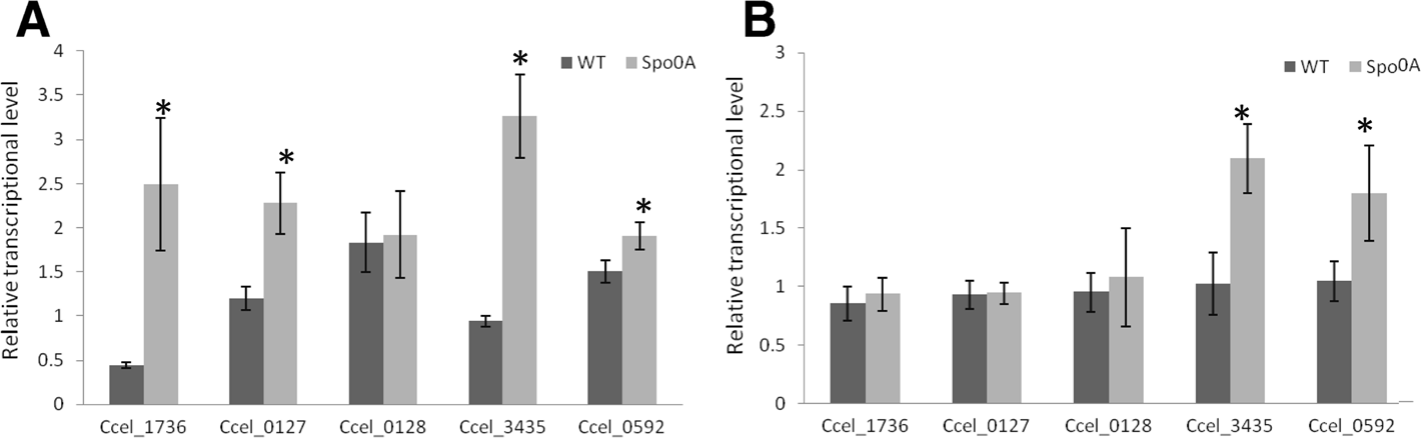
**Gene expression levels by quantitative real-time PCR.** Relative expression level of the selected genes *Ccel_1736*, C*cel_0127*, *Ccel_0128*, *Ccel_3435*, and *Ccel_0592* in wild-type (WT) and *spo0A* mutant grown on 10 g/l cellulose **(A)** and 5 g/l cellobiose **(B)** were compared by normalizing with the expression of the calibrator gene *recA*. The means and standard deviations were calculated from the values of three biological replicates. *Significant difference between WT and *spo0A* mutant (*P* <0.05, Student *t*-test).

Since Spo0A is thought to function as a master transcriptional regulator in other clostridia species [20,21], the global transcriptional profiles of the *spo0A* mutant and WT were compared at the log phase in both defined cellobiose and cellulose media. Compared with the WT strain, a small number of genes with a significant change in expression (Log_2_*R* >2.0; *Z*-score >1.5) were detected in each carbon source, with 87 in cellulose and 141 in cellobiose. Selected genes with expression changes are summarized in detail in Additional file 1. Particularly noticeable were the upregulated genes related to valine, leucine and isoleucine biosynthesis in the *spo0A* mutant, including *Ccel_0127* encoding 3-isopropylmalate dehydratase small subunit, *Ccel_0128* encoding 3-isopropylmalate dehydrogenase, *Ccel_3435* encoding ketol-acid reductoisomerase, and *Ccel_0303* encoding acetolactate synthase (Table 1). To verify the microarray data, five open reading frames (ORFs), including three ORFs in valine, leucine and isoleucine biosynthesis were selected for quantitative reverse transcription PCR analysis. The results showed that the microarray data were closely correlated with the qPCR measurements (Figure 5), except for *Ccel_0128*.

**Table 1:** **Selected genes with significant expression level changes in *spo0A *** **mutant in the category of amino acid transport and metabolism**

	**Cellulose**				**Cellobiose**		
ORF	Annotation	Log_2_*R*	*Z*-score	ORF	Annotation	Log_2_R	Z score
0078	Prephenate dehydratase	4.69	1.60	0303	Acetolactate synthase, large subunit	2.35	4.66
0127	3-Isopropylmalate dehydratase, small subunit	3.69	1.51				
0128	3-isopropylmalate dehydrogenase	3.22	1.98				
3435	Ketol-acid reductoisomerase	3.66	1.77				
3218	Tryptophan synthase, beta subunit	2.50	1.67				
1129	Carboxynorspermidine decarboxylase	2.43	1.77				
0155	Orn/Lys/Arg decarboxylase major region	2.40	1.52				

Genes related to valine, leucine and isoleucine biosynthesis in *spo0A* mutant were detected to be upregulated, including *Ccel_0127* encoding 3-isopropylmalate dehydratase small subunit, *Ccel_0128* encoding 3-isopropylmalate dehydrogenase, *Ccel_3435* encoding ketol-acid reductoisomerase, and *Ccel_0303* encoding acetolactate synthase large subunit. ORF, open reading frame.

To investigate the detailed association between activity changes of metabolic pathways and sporulation inactivation, quantitative gas chromatography–mass spectrometry (GC-MS) analysis was performed to measure changes in a broad range of metabolites found in the culture supernatants. The compounds showing substantial differences in abundance in the mutant versus WT cells are listed in Table 2. Several organic acid levels differed in the *spo0A* mutant and WT cells. Both 2-oxoisovalerate and 2-oxobutyrate were more abundant in the *spo0A* mutant, with concentrations increasing 32-fold and 8-fold relative to the WT cultures, respectively. Both of them are the intermediates in valine, leucine and isoleucine biosynthesis. Thus, the GC-MS data were consistent with the microarray data showing that the expression of *Ccel_0127*, *Ccel_0128*, *Ccel_0303* and *Ccel_3435* was increased. Another noticeable change in GC-MS analysis was the abundance of glucose-6-phosphate (G6P) and glucose-1-phosphate (G1P). The ratio of G6P to G1P is a useful indicator of carbon flux in *C. cellulolyticum*, because these two metabolites represent branch points in hexose metabolism [23]. For WT cells grown in batch cultures the extracellular G6P/G1P ratios were 0.65 at log phase, 3.5 at stationary phase and 7.2 at late stationary phase, as expected due to decreasing growth rates. At all tested growth phases, the G6P/G1P ratios for *spo0A* mutant (0.19, 0.39 and 0.91, respectively) were significantly lower than WT ratios, indicating a change in carbon flux. The increased level of G1P in the *spo0A* mutant cultures could favor the synthesis of glycogen and exopolysaccharides [23].

**Table 2:** **Metabolites differing in abundance between *C. cellulolyticum *** **WT and *spo0A *** **mutant cultures**

	**Wild-type growth phase**			***spo0A *** **growth phase**			
**Compound**^**1**^	**Log**	**Stationary**	**Late stationary**	**Log**	**Stationary**	**Late stationary**	**Sterile medium**
2-Oxoisovalerate	0.32 ± 0.005	0.46 ± 0.03	0.56 ± 0.003	1.75 ± 0.16	18.7 ± 1.78	17.8 ± 0.89	0.08 ± 0.008
2-Oxobutyrate	0.20 ± 0.009	0.17 ± 0.03	0.19 ± 0.002	0.95 ± 0.24	1.63 ± 0.22	1.58 ± 0.19	0.04 ± 0.015
4-Methyl-2-oxovalerate	0.40 ± 0.05	2.43 ± 0.76	1.25 ± 0.24	0.04 ± 0.015	0.07 ± 0.03	0.06 ± 0.02	0.02 ± 0.01
2-Hydroxyglutarate	1.02 ± 0.27	16.51 ± 2.83	26.5 ± 4.12	0.76 ± 0.03	4.20 ± 0.41	3.83 ± 0.27	0.23 ± 0.01
2,3-Dihydroxyisovalerate	1.1 ± 0.27	0.35 ± 0.16	0.04	0.39 ± 0.04	0.15 ± 0.07	0.02 ± 0.006	ND
Malate	9.13 ± 1.39	20.2 ± 2.43	21.7 ± 1.63	3.70 ± 0.40	9.38 ± 0.32	7.59 ± 0.46	0.64 ± 0.05
2,3-Butanediol	0.74 ± 0.16	3.62 ± 0.03	5.13 ± 0.38	1.43 ± 0.24	12.71 ± 1.72	14.3 ± 2.24	0.05 ± 0.005
Glycerol-1phosphate	3.18 ± 0.05	3.36 ± 0.13	4.65 ± 0.72	3.29 ± 0.22	5.85 ± 0.62	6.72 ± 0.40	1.37 ± 0.07
Glucose-6phosphate	24.1 ± 3.76	59.3 ± 13.11	87.0 ± 8.80	9.12 ± 1.40	19.48 ± 6.67	35.3 ± 8.65	ND
Glucose-1phosphate	37.7 ± 8.35	17.3 ± 6.31	12.1 ± 0.18	48.4 ± 7.74	49.8 ± 3.17	38.9 ± 2.94	ND
11.10 min; 246 and 320 *m/z*	5.1 ± 0.28	13.1 ± 2.9	17.8 ± 2.8	0.61 ± 0.07	2.3 ± 0.50	1.43 ± 0.52	0.07 ± 0.02
7.52 min; 159 and 174 *m/z*	1.1 ± 0.007	0.98 ± 0.14	1.07 ± 0.09	2.43 ± 0.25	7.56 ± 0.51	7.0 ± 0.31	0.13 ± 0.03
9.58 min; 117, 259, 244, 288 and 303 *m/z*	0.2 ± 0.03	1.04 ± 0.01	0.52 ± 0.01	0.58 ± 0.11	4.26 ± 1.1	4.66 ± 0.99	0.14 ± 0.03
9.93 min; 331 and 359 *m/z*	11.7 ± 1.2	55.1 ± 11.6	29.6 ± 10.6	0.66 ± 0.18	2.59 ± 1.20	0.61 ± 0.05	0.07 ± 0.006
10.90 min; 450 *m/z*	7.94 ± 0.23	5.48 ± 1.19	3.83 ± 0.34	16.0 ± 1.55	21.6 ± 1.37	16.0 ± 1.55	0.08 ± 0.006

^1^Strains were grown in cellobiose medium. Metabolite concentrations were measured by gas chromatography–mass spectrometry analysis of the derivatized supernatants of duplicate (wild-type) or triplicate (mutant) cultures, as described in the Materials and methods section. The cultures were sampled at 20 hours after growth for log phase, 32 hours for stationary phase, and 48 hours for late stationary phase. Concentrations (μg/ml) are listed as the mean and standard deviation of measurements. Unidentified metabolites are represented by their chromatographic elution times and characteristic peak values. ND, not detected.

### Increased cellulose catabolism by the alleviation of carbon overload

It was previously reported that when *C. cellulolyticum* was grown with high carbon source concentration, high carbon flux was attained, leading to a high level of pyruvate accumulation [8]. As a result, such a catabolic overflow led to an accumulation of intracellular inhibitory compounds that were responsible for the cessation of growth of *C. cellulolyticum*[9]. To further increase cellulose utilization, the excessively accumulated pyruvate needs to be consumed. A biosynthetic strategy was developed to produce higher alcohols by taking advantage of the amino acid biosynthesis capability to produce 2-keto acids, and then the 2-keto acids were converted to alcohols by 2-keto acid decarboxylase and alcohol dehydrogenase [18]. As the valine, leucine and isoleucine biosynthesis pathways were upregulated based on microarray and GC-MS analyses in the *spo0A* mutant, we hypothesized that it could be possible to mitigate the pyruvate accumulation and further increase cellulose consumption by driving the pyruvate flow towards amino acid biosynthesis and further diverting the intermediates toward higher alcohols. Thus, two key genes, *alsS* encoding *B. subtilis* acetolactate synthase and *kivD* encoding *L. lactis* ketoacid decarboxylase, were introduced into both the *spo0A* mutant and WT to direct the conversion of pyruvate to isobutanol.

However, it was shown that strong expression of *alsS* was toxic to *C. cellulolyticum*, because no transformants could be obtained when the *alsS* gene was cloned directly under the ferredoxin promoter from *Clostridium pasteurianum*[5]. The *C. pasteurianum* ferredoxin promoter was a strong constitutive promoter for *C. cellulolyticum*, and was also recognized by *Escherichia coli*[24]. Although this promoter can drive strong expression of its downstream genes, it is uncontrollable in both *C. cellulolyticum* and *E. coli*, causing toxicity in over-expression in *C. cellulolyticum* and cloning in *E. coli*[5]. To mitigate the toxicity of foreign gene over-expression in *C. cellulolyticum*, we decided to utilize inducible promoters recognized by *C. cellulolyticum*.

**Figure 6:**
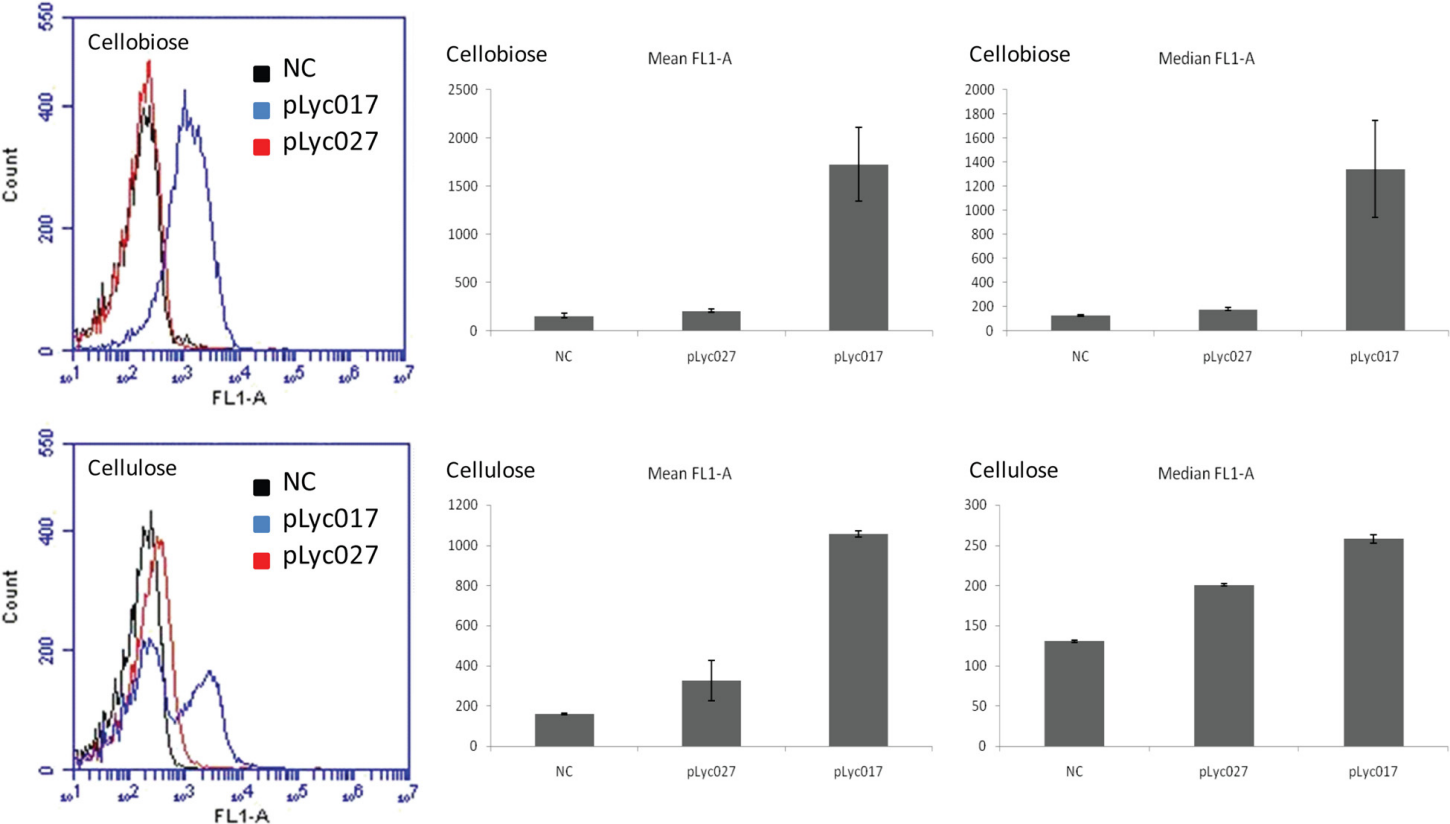
**Flow cytometry data with fluorescent detector channel, FL1-A.** In the cellobiose medium, left upper chart showed an obvious shift of pLyc017 transformants from negative control (NC) with fluorescence intensity, but not for pLyc027 transformants, corresponding to more than 10-fold fluorescent signal intensity increase in the middle upper chart for pLyc017 and only a slightly increase for pLyc027. In the cellulose medium, pLyc017 transformants showed an obviously shifted peak from NC, and pLyc027 transformants also showed a shifted peak (left lower chart). The data also demonstrated that the cipP promoter had different strength in cellobiose and cellulose media, and even with cellulose as the carbon source it was not as strong as the ferrodoxin promoter. The error bars of mean and median FL1-A values represent standard deviations of measurements from three replicate cultures.

**Figure 7:**
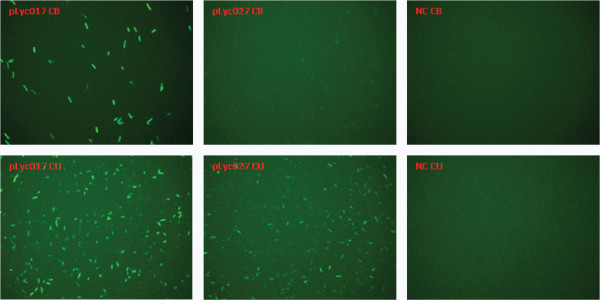
**Microscopic images of cells grown in cellobiose (CB) and cellulose (CU) media.** pLyc017 transformants showed strong fluorescence in both CB and CU media, whereas pLyc027 transformants showed weak fluorescence in CB medium and moderate fluorescence in CU medium.

The promoter tested in this study was cipP, which controls the transcription of the *cip-cel* gene cluster encoding several key components of the cellulosome including the scaffolding protein CipC and major cellulases, including Cel48F, Cel8C and Cel9E, et cetera [25]. Using a transcriptional fusion method, this promoter showed differential strength when *C. cellulolyticum* used cellulose or cellobiose as the carbon source, respectively [26]. For this method, cell lysates needed to be prepared for the chloramphenicol acetyltransferase (CAT) activity assay; therefore, real-time measurement in living cells could not be performed. In the present study, a new protocol was developed for promoter strength evaluation by fusing this promoter with the anaerobic fluorescent protein (AFP) gene Evoglow *Pp1*[27] to achieve real-time monitoring (plasmid pLyc027), and the signal intensity was evaluated by both fluorescent microscopy and flow cytometry. A fusion construction of the *C. pasteurianum* ferredoxin promoter and Evoglow *Pp1* (plasmid pLyc017) was used as a positive control, and the empty vector (pLyc032) was used as a negative control. The flow cytometry data are summarized in Figure 6. When the cipP promoter was cloned in fusion with *Pp1*, cells growing in cellobiose were only able to slightly induce fluorescence expression, a 30% increase compared to the negative control using flow cytometry measurement (shown as mean FL1-A value). When grown in cellulose, the fluorescent signal intensity was increased 106% compared to the negative control (shown as mean FL1-A value). Under fluorescent microscopy, the visibility of the pLyc027 transformants was much more obvious in the cellulose medium than in the cellobiose medium (Figure 7). With both carbon sources, pLyc017 transformants showed strong fluorescence, as demonstrated by more than 10-fold fluorescence increase in cellobiose and more than 6-fold increase in cellulose compared to the negative control, respectively. And even with cellulose as the carbon source, the ferredoxin promoter was much stronger than the cipP promoter. In combination with previously reported data for the CAT activity assay [26], it was concluded that the strength of the cipP promoter was different in cellobiose versus cellulose, with strong stimulation of activity by cellulose. Taken together, the promoter-strength evaluation experiments showed that the cipP promoter transcriptional activity was subjected to regulation, reflecting a preference for cellulose.

**Figure 8:**
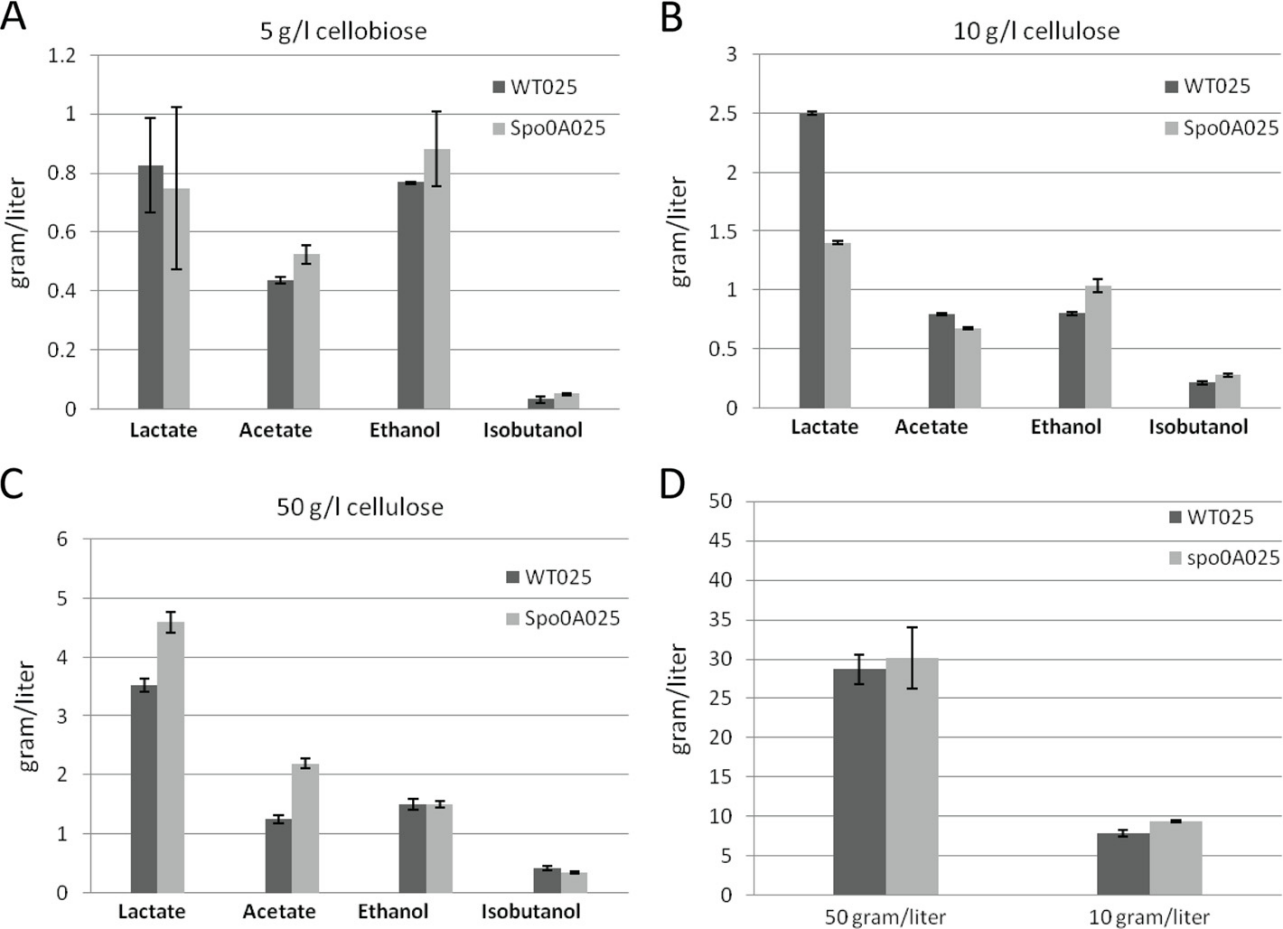
**Final production of lactate, acetate, ethanol and isobutanol and cellulose utilization by *C. cellulolyticum *strains transformed with pLyc025.** The metabolic productivity of the transformants was compared in 5 g/l cellobiose **(A)**, 10 g/l cellulose **(B)** and 50 g/l cellulose **(C)**, respectively. The cellulose utilization at the two different concentrations was also comared **(D)**. The error bars represent standard deviations of measurements from three replicate cultures.

To further increase the cellulose catabolism by alleviating excessive pyruvate accumulation, plasmid pLcy025 was constructed with *Bacillus subtilis alsS* cloned under the cipP promoter followed by *Lactococcus lactis kivD*. This plasmid was transformed successfully into *C. cellulolyticum*. The final metabolite product profiles and cellulose consumption are summarized in Figure 8. In the cellobiose medium, both WT025 and spo0A025 strains produced a negligible amount of isobutanol. In the cellulose medium WT025 consumed 7.84 of 10 g/l cellulose, and spo0A025 consumed 9.36 of 10 g/l. WT025 produced 0.22 g/l isobutanol, and spo0A025 produced 0.29 g/l, a slight increase compared to WT025. With 50 g/l cellulose, the *spo0A* mutant transformed with pLyc025 plasmid did not further increase cellulose consumption, but WT benefited from this carbon overflow alleviation strategy; the introduction of pLyc025 plasmid helped WT increase the cellulose consumption from 17.6 g/l to 28.7 g/l. Unexpectedly, both WT025 and spo0A025 strains did not produce much isobutanol, 0.42 g/l and 0.35 g/l, respectively.

## Discussion

Although sporulation abolishment helped *C. cellulolyticum* improve cellulose utilization and final metabolic product yields in a high concentration of 50 g/l cellulose, the introduction of the partial synthetic isobutanol pathway to consume extra pyruvate did not further increase cellulose utilization in the *spo0A* mutant. One possible explanation is that a connection between sporulation rate and the availability of amino acids in *B. subtilis* was reported [28], and our omics analyses also showed that valine, leucine and isoleucine biosynthesis pathways were altered in the *spo0A* mutant, indicating sporulation abolishment could probably affect amino acid biosynethsis. The defined media used for the fermentation experiments contained salts, vitamins, minerals, nitrogen and carbon without providing additional amino acids. Therefore, the synthetic pathway might cause a valine or leucine deficient environment during the fermentation. Somehow, such a deficiency had a more severe effect on the *spo0A* mutant than WT, resulting in unchanged cellulose utilization. However, the introduction of pLyc025 helped WT increase 63% in cellulose utilization. More efforts will be required to clarify what happened to the carbon flux from the additionally consumed cellulose, since the isobutanol production in WT025 was quite low and the concentrations of the other end-products were not significantly altered. To our surprise, in 50 g/l cellulose, the isobutanol production in spo0A025 was even lower than WT025. Although omics analyses showed upregulation of the valine, leucine and isoleucine biosynthesis pathways in the *spo0A* mutant, the concentration of isobutanol, however, did not show significant increase in the *spo0A* mutant compared to WT, indicating that additional studies are required to connect omics analyses with synthetic pathway regulations. One possibility for the lower than expected isobutanol production is that several paralogous genes exist in the genome, and upregulation of one gene may not necessarily affect the chemical reactions catalyzed by several proteins. For example, there are two genes encoding acetolactate synthase large subunit, *Ccel_0303* and *Ccel_3437*. Only *Ccel_0303* was detected to be up regulated in microarray analysis, but not for *Ccel_3437*. It is also noticeable that the isobutanol productivity was low in both WT and *spo0A* transformants in all tested conditions. Several possible reasons could explain this problem. First, although *alsS* was expressed directly under the cipP promoter, this promoter was relatively less powerful than the ferrodoxin promoter, so that there may not be adequate expression of the two genes, resulting in low productivity. Second, the pathway itself was incomplete and far from optimized. We avoided the inclusion of *ilvC*, *ilvD* and *adh* genes in the construction mainly because the problems of the codon usage of these genes in *C. cellulolyticum* and multiple gene expression have not yet been solved [5].

A key technical achievement of this study was that a quantitative promoter evaluation method was developed using AFP and flow cytometry. It is well known that proper promoters are key elements in metabolic engineering and synthetic biology. Reporter genes, such as those encoding green fluorescent proteins (GFPs) and luciferase, are widely used for the analysis of promoter activities and transcriptional regulation events [29]. However, they all need oxygen for fluorescence and bioluminescence [27], limiting their applications in anaerobic conditions. The recently engineered oxygen-independent flavin mononucleotide (FMN)-based AFPs (Evoglow series), paved the way for many applications associated with GFPs previously unavailable for anaerobic bacteria, such as *in vivo* fluorescence imaging, fluorescence-activated flow cytometry and cell sorting. A protocol for promoter screening was developed using the AFP fusion method, and the fluorescence signal intensity was evaluated by flow cytometry and microscopy. To our knowledge, this is the first report of a successful flow cytometry application with AFP, which holds great potential for further development as a high-throughput promoter screening method using flow cytometry and cell sorting. AFP can also be used as an expression reporter assay; the gene of interest is either transcriptionally or translationally fused to AFP so that the expression level of AFP correlating either to the level of transcription or translation can be quantitatively measured by flow cytometry. Using this promoter evaluation protocol, the strength of the ferrodoxin promoter and cipP promoter was compared. Transformants with the pLyc027 plasmid showed differential fluorescent signal response with cellobiose and cellulose, and the transformants with the pLyc017 plasmid had strong signal intensity with both carbon sources.

## Conclusions

In this study, we presented two metabolic engineering strategies to improve cellulose utilization in *C. cellulolyticum*. The *spo0A* mutant strain abolished sporogenesis and likely became less sensitive to the environmental stress generated by fermentation. In order to alleviate carbon overload, *alsS* and *kivD* genes were introduced to divert the excessive carbon to isobutanol production. Both strategies helped WT significantly increase cellulose utilization under high cellulose concentration.

## Materials and methods

### Media and culture conditions

*E. coli* TOP10 cells (Invitrogen, Grand Island, NY, USA) were used for cloning and were grown at 37°C in LB medium supplemented with 50 μg/ml kanamycin or 20 μg/ml chloramphenicol, as appropriate. *C. cellulolyticum* H10 was cultured at 34°C anaerobically in modified VM medium [5] with 5.0 g/l cellobiose, and 10.0 g/l or 50.0 g/l cellulose as the carbon sources. The complex modified VM medium was supplemented with 2.0 g/l yeast extract and was mainly used for transformation experiments. The defined modified VM medium was supplemented with the vitamin solution and mineral solution as previously described [5], instead of yeast extract and was used for fermentation and omics experiments. For agar plates, 1.0% (w/v) of Bacto agar was added to the medium. The modified VM medium was prepared anaerobically and was supplemented with 15 μg/ml erythromycin or 15 μg/ml thiamphenicol, as appropriate.

### Plasmid construction

The knock-out plasmid pLyc1217Er was constructed as previously described [30]. The intron integration sites were chosen by calculating all possible sites for insertions into *Ccel_1894* using an online intron design tool at http://www.clostron.com[31]. The program predicted multiple intron insertion sites across the gene. Based on the consideration of both optimal gene inactivation and efficient insertion, an anti-sense integration site 60 bp downstream of the start codon was chosen for *Ccel_1894*. Four PCR primers for this integration design, IBS, EBS1d, EBS2 and EBSu were created by the online intron design tool. The knockout plasmid named pLyc1217Er1894 was constructed as previously described [30].

The over-expression plasmids were constructed with pJIR750ai as the backbone by removing the promoter, group II intron and *ltrA*. The ferrodoxin promoter was amplified from pLyc1217Er; the *cipP* promoter was amplified from *C. cellulolyticum* genomic DNA; the AFP gene *Pp1* was amplified from pGLOW-Pp1-stop (The evoglow basic kit, Evocatal, Monheim am Rhein, Germany); *alsS* was amplified from pSA69 and *kivD* was amplified from pSA55 [32]; the *spo0A* gene was amplified from *C. cellulolyticum* genomic DNA. The empty vector was constructed by cutting pJIR750ai with EcoRI and PvuI. The DNA fragments were linked together by standard cloning procedures to generate plasmids, pLyc017, pLyc025, pLyc027, pLyc032, and pSpo0A/over, respectively. A list of primers, plasmids and strains used in this study is presented in Additional file 2.

### Transformation

The plasmids were transformed into *C. cellulolyticum* by electroporation. The electroporation-competent cells and methylated plasmids were prepared as previously described [30]. For each transformation, a 100-μl cell suspension was mixed with 2.0 μg of methylated plasmid DNA. The cells were electroporated in 2-mm gap electroporation cuvettes (BTX, Hawthorne, NY, USA) with a Gene Pulser Xcell electroporator (Bio-Rad, Hercules, CA, USA) inside an anaerobic chamber. The competent cells were transformed by a square wave protocol: the voltage was 1.25 kV, and the time constant was 5 ms. After electroporation, the cells were recovered and plated as previously described [30], and supplemented with 15 μg/ml erythromycin or 15 μg/ml thiamphenicol as appropriate. The plates were incubated at 34°C anaerobically in BD GasPak plastic bags until single colonies appeared.

### Fermentation experiments

All fermentation experiments were run in the defined media with cellobiose or Avicel PH101 crystalline cellulose (FMC BioPolymer, Philadelphia, PA, USA) as the carbon source. Cultures were initially grown in 10 ml of cellobiose medium to an optical density (OD)_600_ of 0.7 to 0.9. These cultures were then used to inoculate either 10 ml cellobiose medium (5.0 g/l) or 100 ml cellulose medium (10 g/l or 50 g/l) at 0.5% (v/v) with three biological replicates for each strain. In 50 g/l cellulose fermentation, pH was adjusted to 7.4 by injecting 5 M NaOH every 48 hours. The pH was monitored by a Cardy Twin micro pH meter (Spectrum Technologies, Aurora, IL, USA). The NaOH injection volumes ranged from 50 μl to 600 μl, depending on the pH values measured before and after injections. The cellular growth was estimated by total protein measurement. The cells were lysed by 0.2 N NaOH/1% w/v SDS for 60 minutes at room temperature, and followed by neutralization with 0.8 N HCl. The total protein was measured with the BCA Protein Assay Kit (Pierce, Rockford, IL, USA), using bovine serum albumin as a standard. The cellulose consumption was determined with glucose as the standard as previously described [9] with minor modifications. The residual cellulose was washed and suspended in distilled water. For attached cell lysis, the suspension was heated at 100°C for 30 minutes. The residual cellulose was further washed with distilled water and hydrolyzed into soluble sugars with 65% H_2_SO_4_. An aliquot of a 200-μl sample was mixed with 200 μl 5% phenol and 1,000 μl 98% H_2_SO_4_ and incubated for 30 minutes at room temperature. Absorbance at 490 nm was determined by a FLUOstar OPTIMA microplate reader (BMG Labtech, Cary, NC, USA), as described previously [33]. For fermentation product analyses, the samples were filtered through 0.2-μm filters, acidified by 0.025% sulfuric acid and analyzed for lactate, acetate, ethanol, and isobutanol concentrations using HPLC with an Agilent 1200 system (Agilent Technologies, Santa Clara, CA, USA) equipped with a variable-wavelength (190 to 600 nm) detector (with UV absorption measured at 245 nm) and an ion-exclusion column (Aminex HPX-87H; 300 mm × 7.8 mm; Bio-Rad, Hercules, CA, USA) operating at 55°C [33]. The mobile phase consisted of 0.025% sulfuric acid at a flow rate of 0.6 ml/minute.

### Southern blotting

*C. cellulolyticum* genomic DNA was extracted using a Wizard Genomic DNA Purification Kit (Promega, Madison, WI, USA): 10 μg of genomic DNA was digested with *EcoRI*, which does not cut the inserted intron fragment, and was separated by agarose gel electrophoresis. The DNA transfer, cross-link, hybridization and detection experiments were performed as previously described [30].

### Heat-survival assay for sporulation

*C. cellulolyticum* strains were grown in defined VM cellulose medium (50 g/l) for 18 days to allow spores to form. Cells were harvested by centrifugation, and suspended and diluted appropriately in anaerobic PBS buffer. Cultures were heated at 80°C for 10 minutes to kill vegetative cells, followed by plating on cellobiose (5.0 g/l) agar plates. Sporulation frequency was calculated as colony-forming unit(cfu)/ml enumerated before and after heat-treatment in triplicates.

### Omics analyses

Transcriptional profile changes were analyzed from triplicate cultures by a whole genome microarray designed and synthesized by NimbleGen (Roche, Madison, WI, USA). DNA microarrays used in this study covered 3078 of the 3390 annotated protein-coding sequences of the *C. cellulolyticum* genome; the probe length was 70-mer and synthesized in triplicate for each gene. Total RNA was collected and isolated for all samples taken at log phase with Trizol and the Qiagen RNA miniprep method as previously described [34]. The fluorescent dye Cyanine 3 was used for labeling of cDNA from total RNA by reverse transcription PCR with random primers. Genomic DNA (gDNA) was labeled with Cyanine 5 and co-hybridized with Cyanine 3-labeled cDNA onto the microarray slide at 42°C on a Hybridization Station (MAUI, BioMicro Systems, Salt Lake City, UT, USA) for 16 h with mixing. Microarray slide washing and image scanning, raw data processing, and fold-change calculation of the gene expression levels were performed as described previously [34,35].

Detailed metabolomic profiles were analyzed from triplicate cultures by GC-MS, using an Agilent Technologies Inc. (Santa Clara, CA, USA) 5975C inert XL gas chromatograph-mass spectrometer, fitted with an Rtx-5MS with Integra-guard (5% diphenyl/95% dimethyl polysiloxane) 30 m × 250 μm × 0.25-μm film-thickness capillary column. Supernatants of *C. cellulolyticum* cultures grown with 5.0 g/l cellobiose were collected at log phase, stationary phase and late stationary phase and centrifuged at 10,000 rpm for 10 minutes at 4°C to remove precipitates. Aliquots containing 250 μl of supernatant and 10 μl of sorbitol (1.0 g/L aqueous) were transferred by pipette to a vial and stored at -20°C overnight. The samples were thawed and concentrated to dryness under a stream of N_2_. The internal sorbitol standard was added to correct for subsequent differences in trimethylsilyl derivatization efficiency and changes in sample volume during heating. Three replicate samples at each phase were analyzed per microbial strain as previously described [36]. Briefly, the standard quadrupole GC-MS was operated with splitless injection and analyses were conducted in the electron impact (70 eV) ionization mode, with 6 full-spectrum (50 to 650 Da) scans per second.

### Real-time PCR quantification

In order to validate microarray hybridization results, five genes were selected for further analysis with real-time PCR. Reverse transcription was conducted by using SuperScript® III Reverse Transcriptase (Invitrogen, Grand Island, NY, USA). cDNA products were diluted as appropriate and used as the templates. Quantitative real-time PCR was performed using iTaq SYBR Green Supermix with ROX (Bio-Rad, Hercules, CA, USA) on Bio-Rad iQ5. Gene-specific primers used for transcript quantification are listed in Additional file 2. The thermal cycling conditions were as follows: 95°C for 3 minutes, 40 cycles of 95°C for 15 s, 55°C for 15 s and 72°C for 45 s. The *recA* gene was used as an internal calibrator [37]. Relative expressional level was calculated with the Pfaffl Method [38].

### Microscopy and flow cytometry

Promoter strength was evaluated by fluorescent microscopy and flow cytometry. Transformants were grown to middle log phase. Samples were washed twice with anaerobic PBS buffer and suspended in the same buffer as well. Slides were imaged using Olympus BX51 fluorescence microscope equipped with an Olympus DP71 digital camera; the optical filter was set with excitation at 490 nm and emission at 525 nm for the green fluorescence. Flow cytometry analysis was performed on a BD Accuri™ C6 flow cytometer (BD Biosciences, San Jose, CA, USA). All samples were diluted with anaerobic PBS buffer approximately 10^6^ to 10^8^ times from the original cultures to similar concentrations. The run limit was set up as 10,000 events with slow flow rate. The threshold was set up as 40,000 on FSC-H. The samples were run through the flow cytometer automatically following the manufacturer’s instructions. The fluorescence was detected with an FL1 detector with a 530/30 filter. The data were collected and analyzed with the CFlow software.

## Abbreviations

AFP: anaerobic fluorescent protein; bp: base pairs; CAT: chloramphenicol acetyltransferase; CBP: consolidated bioprocessing; FMN: flavin mononucleotide; G1P: glucose-1-phosphate; G6P: glucose-6-phosphate; GC-MS: gas chromatography–mass spectrometry; GFP: green fluorescent protein; HPLC: high performance liquid chromatography; ORF: open reading frame; PBS: phosphate-buffered saline; WT: wild-type.

## Competing interests

The authors declare that they have no competing interests.

## Authors’ contributions

YL performed the work presented herein and drafted the manuscript. TX assisted in performing the experiments and analyzing the data. TJT and NLE planned and executed metabolomic experiments and analyzed the results. YY, DEG, ZH and JZ planned experiments, analyzed results and helped draft the manuscript. All authors reviewed and approved the final manuscript.

## Supplementary Material

Additional file 1 **Global transcriptional comparison of the *spo0A *** **mutant and wild-type (WT) using microarray analyses.** This file contains selected genes with significant expression level changes in the *spo0A* mutant. The cells were grown in 10 g/l cellulose or 5 g/l cellobiose to log phase, respectively, in triplicates. Click here for file

Additional file 2 **Primers, plasmids and strains used in this study.** This file contains a list of primers, plasmid vectors and bacterial strains used in this project, along with a list of relevant features or genotypes. Click here for file
