# Supplementary material for: Improvement of cellulose catabolism in Clostridium cellulolyticum by sporulation abolishment and carbon alleviation
Source: Biotechnol Biofuels. 2014 Feb 20;7:25. doi: 10.1186/1754-6834-7-25 (PMC3936895; doi:10.1186/1754-6834-7-25)
Supplement: Additional file 1 — Global transcriptional comparison of the spo0A mutant and wild-type (WT) using microarray analyses. This file contains selected genes with significant expression level changes in the spo0A mutant. The cells were grown in 10 g/l cellulose or 5 g/l cellobiose to log phase, respectively, in triplicates. [file 1754-6834-7-25-S1.docx]

|  |  | Cellulose |  |  |  | Cellobiose |  |  |
| --- | --- | --- | --- | --- | --- | --- | --- | --- |
| Functional category | ORF | Annotation | Log_2_R | Z score | ORF | Annotation | Log_2_R | Z score |
| Energy production  and conversion |  |  |  |  |  |  |  |  |
|  | 1736 | Oxaloacetate decarboxylase | 6.06 | 1.57 | 1738 | Carboxyl transferase | 3.30 | 6.31 |
|  | 0667 | Indolepyruvate ferredoxin oxidoreductase, alpha subunit | 3.50 | 1.53 | 0212 | Phosphoenolpyruvate carboxykinase | 3.17 | 6.12 |
|  | 2503 | Aldo/keto reductase | 3.26 | 1.74 | 2304 | Respiratory-chain NADH dehydrogenase domain 51 kDa subunit | 3.10 | 5.82 |
|  | 3371 | NADH/Ubiquinone/plastoquinone (complex I) | 2.76 | 1.87 | 0902 | Rubrerythrin | 3.02 | 5.87 |
|  | 0934 | Acetyl-CoA carboxylase | 2.63 | 1.69 | 0322 | NifU-like domain-containing protein | 2.87 | 5.57 |
|  | 0553 | 4Fe-4S ferredoxin iron-sulfur binding domain protein | 2.62 | 1.74 | 2305 | NADH dehydrogenase (ubiquinone) 24 kDa subunit | 2.70 | 5.17 |
|  | 3220 | Aldo/keto reductase | 2.08 | 2.02 | 1869 | Rubrerythrin | 2.68 | 4.96 |
|  |  |  |  |  | 0893 | Rubrerythrin | 2.61 | 5.07 |
|  |  |  |  |  | 2303 | Hydrogenase, Fe-only | 2.46 | 4.68 |
|  |  |  |  |  | 1391 | Methylglyoxal synthase | 2.01 | 3.97 |
|  |  |  |  |  | 1359 | 4Fe-4S ferredoxin iron-sulfur binding domain protein | -2.17 | -4.31 |
| Cell division |  |  |  |  |  |  |  |  |
|  | 2559 | Septum site-determining protein MinC | 2.50 | 1.61 | 0475 | MraZ protein | 2.43 | 4.77 |
|  |  |  |  |  | 2907 | Cell division ATP-binding protein FtsE | -2.27 | -4.40 |
| Amino acid transport and metabolism |  |  |  |  |  |  |  |  |
|  | 0078 | Prephenate dehydratase | 4.69 | 1.60 | 1422 | Antifreeze protein type I. Membrane protease subunit, stomatin/prohibitin family | 2.47 | 4.78 |
|  | 0127 | 3-Isopropylmalate dehydratase, small subunit | 3.69 | 1.51 | 0303 | Acetolactate synthase, large subunit, biosynthetic type | 2.35 | 4.66 |
|  | 0128 | 3-isopropylmalate dehydrogenase | 3.22 | 1.98 |  |  |  |  |
|  | 3435 | Ketol-acid reductoisomerase | 3.66 | 1.77 |  |  |  |  |
|  | 3218 | Tryptophan synthase, beta subunit | 2.50 | 1.67 |  |  |  |  |
|  | 1129 | Carboxynorspermidine decarboxylase | 2.43 | 1.77 |  |  |  |  |
|  | 0155 | Orn/Lys/Arg decarboxylase major region | 2.40 | 1.52 |  |  |  |  |
| Nucleotide transport and metabolism |  |  |  |  |  |  |  |  |
|  | 3381 | Purine nucleoside phosphorylase | 4.27 | 1.55 | 1358 | Purine or other phosphorylase family 1 | -2.47 | -4.91 |
|  | 2419 | Adenylosuccinate synthetase | 3.63 | 1.70 | 0674? | competence/damage-inducible protein CinA | 3.41 | 6.37 |
|  | 0394 | IMP dehydrogenase/GMP reductase | 3.33 | 1.62 |  |  |  |  |
|  |  |  |  |  |  |  |  |  |
| Carbohydrate transport and metabolism |  |  |  |  |  |  |  |  |
|  | 1463 | CDP-alcohol phosphatidyltransferase | 7.91 | 3.53 | 2245 | HPrNtr domain-containing protein | 3.79 | 5.95 |
|  | 2111 | Binding-protein-dependent transport systems inner membrane component | 5.08 | 1.55 | 2394 | Major facilitator superfamily MFS_1 | 3.62 | 5.89 |
|  | 1640 | Sugar-phosphate isomerase, RpiB/LacA/LacB family | 3.61 | 4.87 | 2109 | Glycosyltransferase 36 | 3.48 | 6.89 |
|  |  |  |  |  | 2111 | binding-protein-dependent transport systems inner membrane component | 3.11 | 6.10 |
|  | 3407 | UTP-glucose-1-phosphate uridylyltransferase | 3.47 | 1.75 | 3224 | Alcohol dehydrogenase GroES domain protein | 2.49 | 4.92 |
|  | 1431 | Aldose 1-epimerase | 2.66 | 2.25 | 2909 | ABC transporter related | 2.46 | 4.82 |
|  |  |  |  |  | 0806 | Phosphocarrier, HPr family | 2.45 | 4.71 |
|  |  |  |  |  | 1253 | Binding-protein-dependent transport systems inner membrane component | 2.15 | 3.93 |
|  |  |  |  |  | 1671 | PfkB domain protein | -2.09 | -4.15 |
|  |  |  |  |  | 3245 | Binding-protein-dependent transport systems inner membrane component | -2.15 | -4.26 |
| Lipid metabolism |  |  |  |  |  |  |  |  |
|  | 0681 | Fatty acid/phospholipid synthesis protein PlsX | 2.93 | 1.66 | 2887 | 3-Oxoacyl-(acyl-carrier-protein (ACP)) synthase III domain protein | 3.90 | 7.23 |
|  | 0896 | AMP-dependent synthetase and ligase | 2.67 | 1.62 | 0011 | CDP-diacylglycerol/serine O-phosphatidyltransferase | 3.18 | 5.85 |
|  |  |  |  |  | 0852 | Acyl transferase | 2.27 | 4.45 |
|  |  |  |  |  | 0679 | CDP-diacylglycerol/glycerol-3-phosphate 3-phosphatidyltransferase | 2.19 | 4.33 |
|  |  |  |  |  | 2218 | CoA-substrate-specific enzyme activase | -2.16 | -4.25 |
| Translation, ribosomal structure and biogenesis |  |  |  |  |  |  |  |  |
|  | 2134 | Ribosomal protein L32 | 4.37 | 1.63 | 0311 | Ribosomal protein L7/L12 | 4.72 | 8.36 |
|  | 0081 | Ribosomal protein L9 | 3.70 | 2.52 | 1373 | Threonyl-tRNA synthetase | 5.80 | 8.91 |
|  | 3454 | Leucyl-tRNA synthetase | 3.22 | 1.56 | 0710 | Ribosomal protein S16 | 3.60 | 6.97 |
|  | 0445 | Ribosome recycling factor | 2.92 | 1.71 | 3375 | Ribosomal protein L31 | 3.27 | 5.84 |
|  | 0795 | Ribosomal protein S9 | 2.81 | 1.76 | 2094 | Ribosomal protein S21 | 3.04 | 5.97 |
|  | 0314 | Ribosomal protein L7Ae/L30e/S12e/Gadd45 | 2.70 | 1.86 | 0775 | Ribosomal protein S5 | 2.51 | 4.89 |
|  | 1855 | Isoleucyl-tRNA synthetase | 2.63 | 1.83 | 0309 | Ribosomal protein L1 | 2.28 | 4.50 |
|  | 2278 | tRNA/rRNA methyltransferase | 2.06 | 1.56 | 0317 | Translation elongation factor G | 2.11 | 4.06 |
|  |  |  |  |  | 1322 | Ribosomal protein L27 | 2.09 | 4.11 |
| Transcription |  |  |  |  |  |  |  |  |
|  | 0592 | Transcriptional regulator, MerR family | 2.92 | 1.86 | 1905 | NusB antitermination factor | 3.21 | 6.25 |
|  | 1228 | Two component transcriptional regulator, AraC family | 2.46 | 4.65 | 2424 | Histone family protein DNA-binding protein | 3.04 | 5.49 |
|  | 3487 | Single-stranded nucleic acid binding R3H domain protein | 2.36 | 1.60 | 1460 | Tryptophan RNA-binding attenuator protein | 2.71 | 5.10 |
|  |  |  |  |  | 0592 | Transcriptional regulator, MerR family | 2.70 | 5.19 |
|  |  |  |  |  | 2924 | Transcriptional modulator of MazE/toxin, MazF | 2.56 | 4.97 |
|  |  |  |  |  | 1790 | DNA-directed RNA polymerase, omega subunit | 2.35 | 4.65 |
|  |  |  |  |  | 1857 | Transcriptional regulator, GntR family with aminotransferase domain | -2.04 | -4.06 |
| DNA replication, recombination and repair |  |  |  |  |  |  |  |  |
|  | 0082 | Replicative DNA helicase | 3.95 | 1.76 | 1751 | Tyrosine recombinase XerD | 2.89 | 5.59 |
|  | 1882 | ATP-dependent DNA helicase RecG | 3.65 | 1.63 | 3059 | DNA methylase N-4/N-6 domain protein | 2.58 | 5.02 |
|  | 0041 | IstB domain protein ATP-binding protein | 2.87 | 1.62 | 3050 | Putative phage terminase, large subunit | 2.51 | 4.89 |
|  | 3329 | 8-Oxoguanine DNA glycosylase domain protein | 2.81 | 1.82 | 1867 | Excinuclease ABC C subunit domain protein | 2.50 | 4.83 |
|  | 1903 | Exodeoxyribonuclease VII, small subunit | 2.40 | 1.66 | 3078 | Resolvase domain protein | 2.16 | 4.15 |
|  |  |  |  |  | 1566 | Replication initiation factor | -2.28 | -4.48 |
| Cell motility and secretion |  |  |  |  |  |  |  |  |
|  | 2053 | Flagellar basal-body rod protein FlgC | 3.74 | 1.52 | 0091 | Regulatory protein, MerR | 3.84 | 6.58 |
|  | 0094 | Flagellar hook-associated protein FlgK | 2.24 | 1.70 | 0100 | Flagellin domain protein | 3.56 | 6.98 |
|  | 0093 | FlgN family protein | 2.18 | 1.73 |  |  |  |  |
| Posttranslational modification, protein turnover, chaperons |  |  |  |  |  |  |  |  |
|  |  |  |  |  | 1798 | Chaperone protein DnaK | 3.42 | 6.65 |
|  |  |  |  |  | 0391 | Chaperonin Cpn10 | 2.96 | 5.58 |
|  |  |  |  |  | 1876 | Cysteine desulfurase family protein | 2.59 | 5.10 |
| Inorganic ion transport and metabolism |  |  |  |  |  |  |  |  |
|  | 0296 | ABC-2 type transporter | 3.06 | 2.20 | 1998 | FeoA family protein | 2.96 | 5.71 |
|  | 0746 | Copper-translocating P-type ATPase | 2.80 | 1.70 | 0436 | Binding-protein-dependent transport systems inner membrane component | 2.61 | 4.98 |
|  |  |  |  |  |  |  |  |  |
| Secondary metabolites biosynthesis, transport and catabolism |  |  |  |  |  |  |  |  |
|  | 1151 | ABC transporter related | 3.87 | 1.67 | 3261 | ABC-2 type transporter | -2.16 | -4.28 |
|  | 1872 | Silent information regulator protein Sir2 | 3.72 | 1.67 |  |  |  |  |
|  |  |  |  |  |  |  |  |  |
| Signal transduction mechanisms |  |  |  |  |  |  |  |  |
|  | 2037 | Response regulator receiver protein | 2.93 | 1.74 | 0049 | Methyl-accepting chemotaxis sensory transducer | 5.65 | 10.33 |
|  | 3117 | Signal transduction histidine kinase regulating citrate/malate metabolism | -2.04 | -3.71 | 0497 | Metal dependent phosphohydrolase | 2.19 | 4.25 |
|  |  |  |  |  | 2027 | CheA signal transduction histidine kinase | 2.18 | 4.20 |
|  |  |  |  |  | 1382 | band 7 protein | 2.02 | 3.94 |
| Intracellular trafficking, secretion, and vesicular transport |  |  |  |  |  |  |  |  |
|  |  |  |  |  | 0536 | Protein-export membrane protein SecD | 2.74 | 5.25 |
| Cellulosome related |  |  |  |  |  |  |  |  |
|  | 0739 | Cellulosome protein dockerin type I | 4.04 | 1.68 | 0429 | PKD domain containing protein | -2.65 | -5.18 |
|  | 1207 | Cellulosome protein dockerin type I | 3.92 | 1.53 | 2226 | Glycoside hydrolase family 9 | -2.72 | -5.38 |
|  | 1648 | Glycoside hydrolase family 9 | 3.87 | 1.85 | 1866 | Glycoside hydrolase family 11 | -2.88 | -4.69 |

Changes in the expression of genes encoding ribosomal proteins were found in both growth conditions and all of them were up-regulated, indicating that the disruption of sporulation affected the cell's protein synthesis machinery. Several genes encoding flagellar assembly were up-regulated in the *spo0A* mutant, including *Ccel_0093*, *Ccel*_*0094*, and *Ccel_2053* in cellobiose, and *Ccel_0091* and *Ccel_0100* in cellulose. The related bacterial chemotaxis pathway genes, such as *Ccel_2037* and *Ccel_2027* were up-regulated accordingly. Similar changes in gene expression related to motility and chemotaxis were also observed in the *C. acetobutylicum* *spo0A* mutant [[1](#_ENREF_1)]. *Ccel_2907* encoding the cell division ATP binding protein FtsE was down-regulated, FtsE directly interacted with the cell division GTPase FtsZ [[2](#_ENREF_2)], and the latter gene was shown to play an essential role in cell division and sporulation in *Bacillus subtilis* [[3](#_ENREF_3)]. *Ccel_2559* encoding the cell division inhibitor MinC [[4](#_ENREF_4)] and *Ccel_0475* encoding the MraZ protein playing a role in cell-wall biosynthesis and cell division [[5](#_ENREF_5)] were up-regulated in the *spo0A* mutant. In both cellobiose and cellulose, the expression of a MerR family transcription regulator, *Ccel_0592*, was increased. The MerR regulators mainly respond to environmental stresses, such as oxidative stress, heavy metals or antibiotics [[6](#_ENREF_6)]. A two component AraC family transcriptional regulator, *Ccel_1228* was up-regulated in cellobiose and a GntR family transcription regulator, *Ccel_1857* was down-regulated in cellulose. The expression of several nitrogen metabolism related genes, *Ccel_2303*, *Ccel_2304* and *Ccel_2305* was up-regulated in the *spo0A* mutant, and all these responses were detected in cellulose, but not in cellobiose, indicating a possible connection between sporulation, cellulose catabolism and nitrogen metabolism. Significantly increased expression of genes involved in central pyruvate metabolism, such as *Ccel_0212*, *Ccel_0934* and *Ccel_1736* were detected in the *spo0A* mutant.

1. Tomas CA, Alsaker KV, Bonarius HP, Hendriksen WT, Yang H, Beamish JA, Paredes CJ, Papoutsakis ET: **DNA array-based transcriptional analysis of asporogenous, nonsolventogenic *Clostridium acetobutylicum* strains SKO1 and M5.** *J Bacteriol* 2003, **185:**4539-4547.

2. Corbin BD, Wang YP, Beuria TK, Margolin W: **Interaction between cell division proteins FtsE and FtsZ.** *Journal of Bacteriology* 2007, **189:**3026-3035.

3. Feucht A, Errington J: ***ftsZ* mutations affecting cell division frequency, placement and morphology in *Bacillus subtilis*.** *Microbiology* 2005, **151:**2053-2064.

4. de Boer PA, Crossley RE, Rothfield LI: **Central role for the *Escherichia coli* *minC* gene product in two different cell division-inhibition systems.** *Proc Natl Acad Sci U S A* 1990, **87:**1129-1133.

5. Adams MA, Udell CM, Pal GP, Jia ZC: **MraZ from *Escherichia coli*: cloning, purification, crystallization and preliminary X-ray analysis.** *Acta Crystallogr F* 2005, **61:**378-380.

6. Brown NL, Stoyanov JV, Kidd SP, Hobman JL: **The MerR family of transcriptional regulators.** *FEMS microbiology reviews* 2003, **27:**145-163.
