# Supplementary material for: Improvement of cellulose catabolism in Clostridium cellulolyticum by sporulation abolishment and carbon alleviation
Source: Biotechnol Biofuels. 2014 Feb 20;7:25. doi: 10.1186/1754-6834-7-25 (PMC3936895; doi:10.1186/1754-6834-7-25)
Supplement: Additional file 2 — Primers, plasmids and strains used in this study. This file contains a list of primers, plasmid vectors and bacterial strains used in this project, along with a list of relevant features or genotypes. [file 1754-6834-7-25-S2.docx]

| **Primer name** | **Sequence** | **Function** |
| --- | --- | --- |
| EBSu | CGAAATTAGAAACTTGCGTTCAGTAAAC | EBS universal primer for cross-over PCR |
| Spo0A60aIBS | AAAACCCGGGATAATTATCCTTAGTACTCGCATAAGTGCGCCCAGATAGGGTG | IBS primer for cross-over PCR to retarget *Ccel_1894* |
| Spo0A60aEBS1d | CAGATTGTACAAATGTGGTGATAACAGATAAGTCGCATAAGATAACTTACCTTTCTTTGT | EBS1d primer for cross-over PCR to retarget *Ccel_1894* |
| Spo0A60aEBS2 | TGAACGCAAGTTTCTAATTTCGATTAGTACTCGATAGAGGAAAGTGTCT | EBS2 primer for cross-over PCR to retarget *Ccel_1894* |
| Spo0AF | GGTCTTTAACAACCATCATAATTGC | Forward primer to screen *Ccel_1894* mutant and sequence |
| Spo0AR | GGTTAATGATCCTACGAGAGGGTAT | Reverse primer to screen *Ccel_1894* mutant and sequence |
| pintronF1 | CCTATGGGAACGAAACGAAA | Intron-specific primer to screen and sequence |
| pintronR1 | CGAGTACTCCGTACCCTTGC | Intron-specific primer to screen and sequence |
| FdF | CAAAAGGATCCGCGCCCCGGATCGAGATAGTATATG | Forward primer to amplify the ferredoxin promoter |
| FdR | CAAAAGGATCCTACCGAGCTCGAATTCGTAATCATG | Reverse primer to amplify the ferredoxin promoter |
| CipPF | CAAAACCTGCAGGGTCAAGAAGTAATTACAAGTCCAAT | Forward primer to amplify the CipP promoter |
| CipPR | CAAAAGGATCCCCTCCTATTTTTTATTTTACATGAA | Reverse primer to amplify the CipP promoter |
| AlsSF | CAAAAGGATCCTTGACAAAAGCAACAAAAGAACAAA | Forward primer to amplify *alsS* gene |
| AlsSR | CAAAACTCGAGTTAGAGAGCTTTCGTTTTCATGAGT | Reverse primer to amplify *alsS* gene |
| KivDF | CAAAACTCGAGAGGAGGTTTACAATGTATACAGTAGGAGATTACCTAT | Forward primer to amplify *kivD* gene |
| kivDR | CAAAAGGCGCCTTATGATTTATTTTGTTCAGCAAAT | Reverse primer to amplify *kivD* gene |
| Pp1F | CAAAAGGATCCATGATCAACGCAAAACTCCTGCAAC | Forward primer to amplify *Pp1* gene |
| Pp1R | CAAAAGGCGCCTCAGTGCTTGGCCTGGCCCTGCTGC | Reverse primer to amplify *Pp1* gene |
| Spo0A/overF | CAAAAGGATCCTTGAGTAGCAAAAAAATAGAAGTCC | Forward primer to amplify *spo0A* gene |
| Spo0A/overR | CAAAAGGCGCCTTAGCTTACCTTCAATTCCAATCTC | Reverse primer to amplify *spo0A* gene |
| RTrecAF | GCAAAGAAACTTGGGGTTGA | *recA* qPCR |
| RTrecAR | TGAGACATCAGCCTTGCTTG | *recA* qPCR |
| RT1736F | ACCATTCACTGGAAGCTTGG | *Ccel_1736* qPCR |
| RT1736R | CATGCCTTAATTGCCGTCTC | *Ccel_1736* qPCR |
| RT0127F | GGTTCATCAAGGGAACATGC | *Ccel_0127* qPCR |
| RT0127R | GCCGTCAGCTGATATAATCTCC | *Ccel_0127* qPCR |
| RT0128F | TGATGCAGTTCTCCTTGGTG | *Ccel_0128* qPCR |
| RT0128R | CGAAATACATTCCGCCTGTC | *Ccel_0128* qPCR |
| RT3435F | GGAATACGCTTTGGCATACG | *Ccel_3435* qPCR |
| RT3435R | GTGTTTCAAACCCTGCCTTC | *Ccel_3435* qPCR |
| RT0592F | AGCTTGAACTTGGAGCTTGC | *Ccel_0592* qPCR |
| RT0592R | CAGTTTACCAGCCAGAGATGTG | *Ccel_0592* qPCR |
| **Plasmid** | **Relevant features** | **Source** |
| pJIR750ai | L1.LtrB-ΔORF intron, *ltrA* | Chen Y *et al*. (2005) |
| pLyc1217Er | Fd promoter, L1.LtrB-ΔORF intron, *ltrA* | Li YC *et al.* (2012) |
| pLyc007 | pLyc1217Er with intron retargeting *Ccel_1894* | This study |
| pLyc017 | Cmp^R^, Fd promoter, *Pp1* | This study |
| pLyc027 | Cmp^R^, CipP promoter, *Pp1* | This study |
| pLyc025 | Cmp^R^, CipP promoter, *alsS*, *kivD* | This study |
| pLyc032 | Cmp^R^ pIP404 origin, pMB1 origin | This study |
| pSpo0A/over | Cmp^R^, Fd promoter, *spo0A* | This study |
| **Strain** | **Relevant genotype** | **Source** |
| *E. coli* TOP10 | *recA1* *mcrA hsdR endA1* | Invitrogen |
| WT | Wild-type *C. cellulolyticum* H10 | ATCC 35319 |
| *spo0A* mutant | *Ccel_1894*::LtrB | This study |
| WT017 | Wild-type transformed with pLyc017, Tmp^R^ | This study |
| WT027 | Wild-type transformed with pLyc027, Tmp^R^ | This study |
| WT032 | Wild-type transformed with empty pLyc032, Tmp^R^ | This study |
| WT025 | Wild-type transformed with pLyc025, Tmp^R^ | This study |
| Spo0A025 | *spo0A* mutant transformed with pLyc025, Tmp^R^ | This study |
| Spo0A/over | *spo0A* mutant transformed with pSpo0A/over, Tmp^R^ | This study |
| Spo0A/zero | *spo0A* mutant transformed with empty pLyc032, Tmp^R^ | This study |
